# Cryotherapy Attenuates Inflammation via the lncRNA SNHG1/miR-9-5p/NFKB1 Regulatory Axis in Periodontal Ligament Cells

**DOI:** 10.3390/ijms241512097

**Published:** 2023-07-28

**Authors:** Can Lin, Miaomiao Liu, Jihua Guo, Rong Jia

**Affiliations:** 1 State Key Laboratory of Oral & Maxillofacial Reconstruction and Regeneration, Key Laboratory of Oral Biomedicine Ministry of Education, Hubei Key Laboratory of Stomatology, School & Hospital of Stomatology, Wuhan University, Wuhan 430072, China; lincan@whu.edu.cn (C.L.); miaomiaoliu@whu.edu.cn (M.L.); 2 Department of Endodontics, School & Hospital of Stomatology, Wuhan University, Wuhan 430079, China

**Keywords:** cryotherapy, apical periodontitis, SNHG1, NFKB1

## Abstract

Cryotherapy is a common non-pharmacological method to relieve pain and inflammation. Clinical studies have shown that cryotherapy can reduce postoperative pain after root canal therapy, but the mechanism remains unclear. In this study, we aimed to investigate the underlying molecular mechanisms by which cryotherapy reduces inflammation in lipopolysaccharide (LPS)-stimulated periodontal ligament cells through transcriptome sequencing analysis. We found that cryotherapy significantly reduced the expression of multiple proinflammatory cytokines and chemokines, and NFKB1 was the key regulator down-regulated by cryotherapy. Importantly, we discovered that lncRNA SNHG1 expression level significantly decreased after cold treatment. SNHG1 expression was positively related to NFKB1 while negatively correlated with miR-9-5p, which formed a novel ceRNA regulatory pathway. Knockdown of SNHG1 significantly reduced the expression of NFKB1, IL1B, and IL6, while overexpression of SNHG1 significantly increased the expression of these genes. In conclusion, our study demonstrated that cryotherapy can effectively reduce inflammation in LPS-induced periodontal ligament cells by suppressing the lncRNA SNHG1/miR-9-5p/NFKB1 axis.

## 1. Introduction

Cryotherapy is a method of decreasing the temperature for therapeutic purposes. Cryotherapy has been frequently applied in various fields of medicine, such as relieving sports injuries and postoperative pain by reducing tissue temperature to reduce edema and inflammation [1]. Furthermore, cryotherapy is also used in tumor therapy by inducing cell death, tissue ischemia, and necrosis [2,3]. In the treatment of oral diseases, cryotherapy is used to relieve postoperative pain and swelling after tooth extraction [4,5] and treat gingival hyperplasia in a relatively painless and bleeding-free manner [6]. Meanwhile, oral cryotherapy is effective in preventing or reducing the severity of oral mucositis in patients with cancer treatment [7]. Recent clinical studies have shown that cryotherapy in root canal therapy can reduce postoperative pain and inflammation of apical periodontitis.

Apical periodontitis (AP) is a chronic inflammatory disorder of periradicular tissues mainly developed from persistent microbial infection in the dental pulp [8]. Apical periodontitis, as an infectious oral disease, is mainly caused by the dynamic interaction between the host immune response and microbial infection and leads to localized inflammatory infiltrates, alveolar bone resorption, destruction of other periapical tissues, and even tooth loss [9]. Root canal therapy can effectively control infection and promote periapical tissue healing, which is the current clinical treatment of choice for treating apical periodontitis [10]. However, inflammation recurrence and postoperative pain are common problems caused by residual necrotic content, bacteria, and their metabolites in the root canal system after root canal therapy [11]. Postoperative pain after root canal therapy has been reported with a high incidence rate ranging between 3% and 58% [12]. Therefore, there is an urgent need for novel strategies for improving root canal treatment outcomes.

Multiple randomized clinical trials have demonstrated that cryotherapy is an effective, simple, and cheap method to reduce postoperative pain [13,14]. The cryotherapy applications in patients with pulpal or periapical periodontitis mainly refer to the intracanal cryotherapy in root canal therapy (i.e., using 2.5 °C or 4 °C cold saline solution as final irrigation) [15,16]. Some clinical studies involve intraoral and extraoral cryotherapy with ice packs [17]. Meanwhile, an in vitro study demonstrated cryotherapy also has the potential to result in a local anti-inflammatory effect in periradicular tissues [18]. However, the underlying mechanisms of cryotherapy in treating apical periodontitis remain largely unknown.

In the present study, we performed transcriptome analyses by RNA-Seq to identify the differentially expressed genes (DEGs) of lipopolysaccharide (LPS)-stimulated periodontal ligament cells (PDLCs) with or without cryotherapy. A lncRNA-miRNA-mRNA network analysis was conducted to explore the potential roles between differentially expressed lncRNAs and mRNAs. Then, further investigation showed that cryotherapy could inhibit LPS-induced inflammation by regulating the lncRNA SNHG1/miR-9-5p/NFKB1 axis. We aimed to gain insight into the molecular mechanisms of decreasing postoperative pain and inflammation after cryotherapy and to provide a theoretical basis for clinical trials.

## 2. Results

### 2.1. Cryotherapy Reduces LPS-Induced Inflammatory Response in hPDLCs

**Figure 1:**
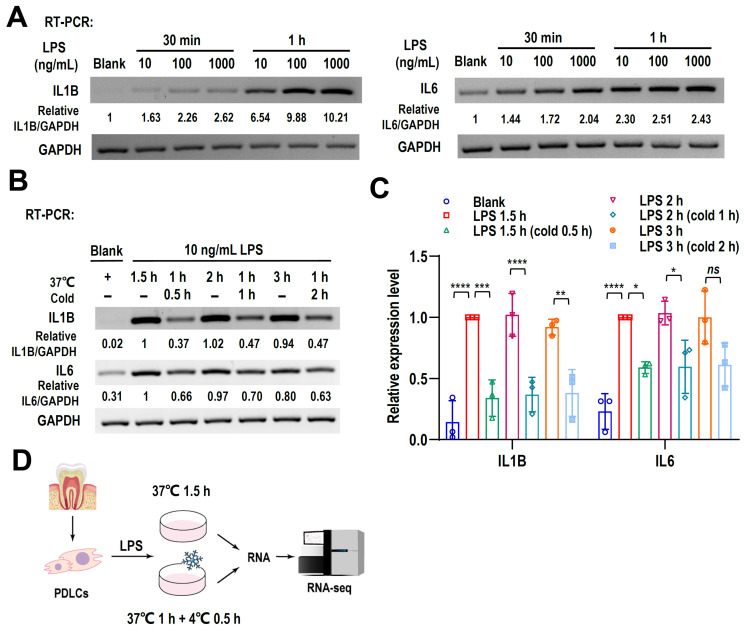
Cryotherapy reduces LPS-induced inflammatory response in hPDLCs. (**A**) The expression of IL1B and IL6 in LPS-stimulated hPDLCs was analyzed by RT-PCR. Blank is the no LPS treatment control. (**B**) Expression of proinflammatory cytokines (IL1B, IL6) in LPS-activated hPDLCs with or without cryotherapy was demonstrated by RT-PCR. Blank is the no LPS and cold treatment control. (**C**) The histograms summarized the differences in the expression of IL1B and IL6 after cryotherapy. Data are means ± SD, *n* = 3. (**D**) Flow diagram showing the entire experimental procedure. * *p* < 0.05, ** *p* < 0.01, *** *p* < 0.001, **** *p* < 0.0001, and “ns” menas no significance.

To explore the roles of cryotherapy in reducing periapical inflammation, we used LPS-stimulated human PDLCs. Human PDLCs were stimulated with a concentration (10–1000 ng/mL) gradient of LPS for different durations to induce an inflammatory response. Expression levels of inflammatory biomarkers IL1B and IL6 increased with LPS stimulation time and concentration (Figure 1A and Figure S1A). As expected, low-dose and short-term stimulation of LPS can induce significant inflammation, indicated by the increased IL1B and IL6 (Figure 1A). Therefore, we selected the treatment of 10 ng/mL LPS for at least 1 h to stimulate hPDLCs to construct the periapical inflammation model for the following experiment. Then, the inflammatory hPDLCs were cultured at 4 °C for different amounts of time to observe the effect of cryotherapy treatment on inflammation (Figure S1B and Figure 1B). 20 min cold treatment could reduce the expression of IL1B. However, 30 min cold treatment showed an obvious decrease in IL1B and IL6 compared with the control cells (treated with LPS but without cold treatment), and longer cold treatment (1 h or 3 h) could not further reduce the expression of IL1B and IL6 (Figure S1B,C and Figure 1B,C). Therefore, we chose 30 min cold treatment for our following experiments.

To better understand the molecular mechanism of cryotherapy reducing inflammation, human PDLCs were randomly assigned to LPS-stimulated cells with cold treatment (cold) and LPS-stimulated cells without cold treatment (control) cells. All PDLCs were stimulated with 10 ng/mL LPS. The cold-treated group was cultured at 37 °C for 1 h, and then treated at 4 °C for 0.5 h. The control group was cultured at 37 °C for 1.5 h. Total cellular RNA was extracted for transcriptome analysis. The entire experimental procedure is shown as a flow scheme in Figure 1D.

### 2.2. Profiles of Differentially Expressed lncRNAs and mRNAs in LPS-Stimulated hPDLCs after Cryotherapy

**Figure 2:**
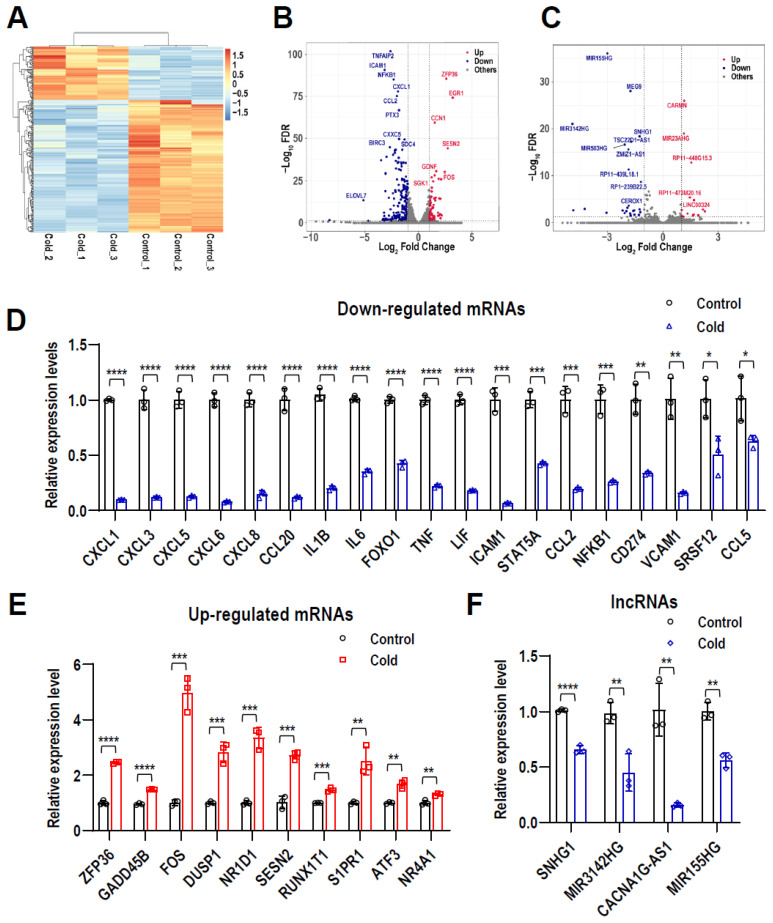
Differentially expressed lncRNAs and mRNAs between LPS-stimulated cells with cold treatment (cold) and LPS-stimulated cells without cold treatment (control). (**A**) Heatmap of differentially expressed genes in each sample. Each row represents a gene, each column represents a sample, and the red color indicates high expression genes, and the blue indicates low expression genes. (**B**,**C**) Volcano plot of differentially expressed lncRNAs (**B**) and mRNAs (**C**). The red dots represent up-regulated DEGs, the blue dots represent downregulated DEGs, and the gray dots represent genes that did not show significant differential expression. (**D**–**F**) Verification of the relative expression levels of mRNA and lncRNAs by qRT-PCR. Data are means ± SD, *n* = 3. * *p* < 0.05, ** *p* < 0.01, *** *p* < 0.001 and **** *p* < 0.0001.

RNA-seq analysis was performed on six samples with three biological replicates per group. The RNA-seq data displayed 287 differentially expressed genes (DGEs) with |log_2_ Fold Change| ≥ 1 and *q*-value ≤ 0.05 (Figure 2A). Among them, a total of 247 mRNAs and 40 lncRNAs were differentially expressed. Compared with the normal control group, the cold-treated group exhibited 14 up-regulated and 26 down-regulated lncRNAs (Figure 2B) and 67 up-regulated and 180 down-regulated mRNAs (Figure 2C). The above results indicated that the gene expression profile after cryotherapy was significantly changed compared with the control group.

To verify the credibility of RNA-Seq data, we randomly selected the coding and lncRNA genes and performed qRT-PCR to verify their relative expression levels in treated or nontreated hPDLCs. As expected, most of them could be verified. *GADD45B*, *ZFP36*, *NR1D1*, *FOS*, *DUSP1*, *SESN2*, *RUNX1T1*, *S1PR1*, *ATF3*, and *NR4A1* were significantly up-regulated, and chemokine family member genes (*CXCL1*, *CXCL3*, *CXCL5*, *CXCL6*, *CXCL8*, *CCL2*, *CCL5*, and *CCL20*), *FOXO1*, *TNF*, *LIF*, *ICAM1*, *STAT5A*, *NFKB1*, *CD274*, *VCAM1*, and *SRSF12* were down-regulated in the cryotherapy group compared with the control group. Meanwhile, the expression levels of lncRNAs, including *SNHG1*, *MIR3142HG*, *CACNA1G-AS1*, and *MIR155HG*, were significantly down-regulated. The relative expression levels of mRNA and lncRNA obtained from the qRT-PCR were correlated with the RNA-seq data, which further verified the credibility of the results (Figure 2D–F).

### 2.3. GO Functional Enrichment Analysis of DEGs

**Figure 3:**
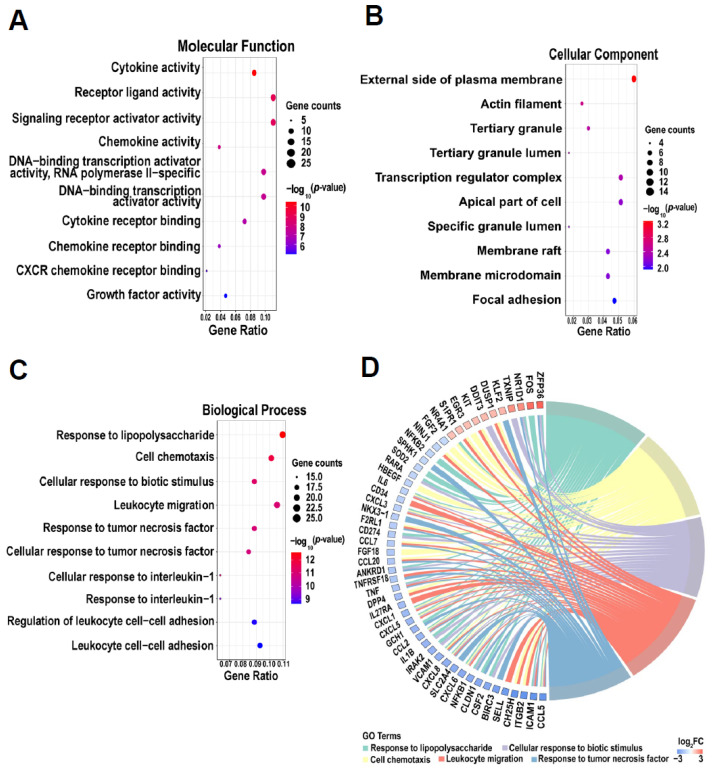
Gene ontology (GO) functional enrichment analysis of DEGs. (**A**–**C**) Bubble plots showing the top 10 significant molecular functions, cellular components, and biological processes associated with DEGs. The dot size represents the number of enriched DEGs, and the color reflects the significance (*p*-value). DEGs are differentially expressed genes between cold-treated and non-cold-treated (control) cells. (**D**) Chord plot showing the connections between DEGs and their assigned GO terms of biological processes via ribbons. Genes are ordered according to the log_2_ Fold Change (log_2_ FC) in the sample. Colored rectangles represent the log_2_ FC of genes.

We performed GO analysis to better understand the biological functions of DEGs, which significantly altered (including both up-regulated and downregulated DEGs) in PDLCs after cryotherapy. The top 10 GO terms were selected from each ontology for visualization according to their *p*-value (Figure 3A–C). Cytokine activity (GO:0005125), receptor-ligand activity (GO:0048018), and signaling receptor activator activity (GO:0030546) were the most enriched molecular functions (MF). The external side of the plasma membrane (GO:0009897), actin filament (GO:0005884), and tertiary granule (GO:0070820) were the most enriched cellular components (CC). Response to lipopolysaccharide (GO:0032496), cell chemotaxis (GO:0060326), and cellular response to biotic stimulus (GO:0071216) were the most enriched biological processes (BP).

The top five GO terms of biological processes were ranked by the number of counts, including “response to lipopolysaccharide”, “cell chemotaxis”, “cellular response to biotic stimulus”, “leukocyte migration”, and “response to tumor necrosis factor” (Figure 3D). The upregulation of *NR4A1*, *NR1D1*, *FOS*, and *ZFP36* was directly related to the response to lipopolysaccharide after cryotherapy. The significantly downregulated chemokine family member genes (*CCL5*, *CXCL6*, *CXCL8*, *CCL2*, *CXCL5*, *CXCL1*, *CCL20*, *CCL7*, and *CXCL3*) were associated with cell chemotaxis and leukocyte migration.

### 2.4. KEGG Enrichment Analysis and GSEA

**Figure 4:**
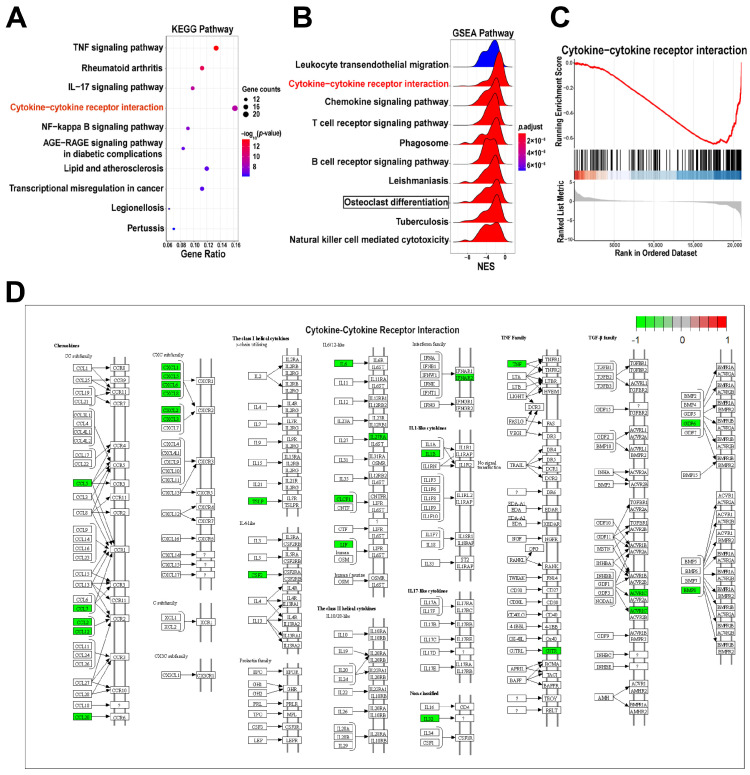
KEGG Enrichment Analysis. (**A**) Bubble plots of the top 10 significantly enriched KEGG pathways. The dot size and color intensity represent the gene count and *p*-value. (**B**) Ridge plots of the top 10 GSEA results. Red indicates a higher significance, and blue indicates a lower significance. (**C**) The GSEA analysis of the cytokine-cytokine receptor interaction pathway. (**D**) The KEGG pathway map of cytokine-cytokine receptor interaction pathway.

KEGG analysis of the DEGs determined that 50 pathways were significantly altered in PDLCs after cryotherapy. The top 10 significantly enriched KEGG pathways showed significant differences in inflammatory pathways, including the TNF signaling pathway, IL-17 signaling pathway, cytokine-cytokine receptor interaction, NF-kappa B signaling pathway, and so on (Figure 4A). Gene set enrichment analysis (GSEA) identified 83 pathways based on NOM *p*-value ≤ 0.05 and FDR *q*-value ≤ 0.25. The top 10 representative pathways with the lowest *p*-value were selected for display, including cytokine-cytokine receptor interaction, chemokine signaling pathway, leukocyte transendothelial migration, and so on (Figure 4B).

Cytokine-cytokine receptor interaction is the only overlapping pathway in both of the top 10 GSEA results and KEGG pathways. The peak in the GSEA result of the cytokine-cytokine receptor interaction pathway was shifted right, with NES = −2.078, indicating that most of the genes in this pathway were downregulated (Figure 4C). Cryotherapy mainly affects the cytokine-cytokine receptor interaction by reducing the molecules related to chemokines, the CXC subfamily, and the TNF family (Figure 4D). Interestingly, the osteoclast differentiation pathway was also significantly affected after cryotherapy (Figure S2). Cryotherapy mainly affects osteoclast differentiation by regulating NFKB/AP-1 signal pathways (Figure S3).

### 2.5. Protein–Protein Interaction Network Analysis

**Figure 5:**
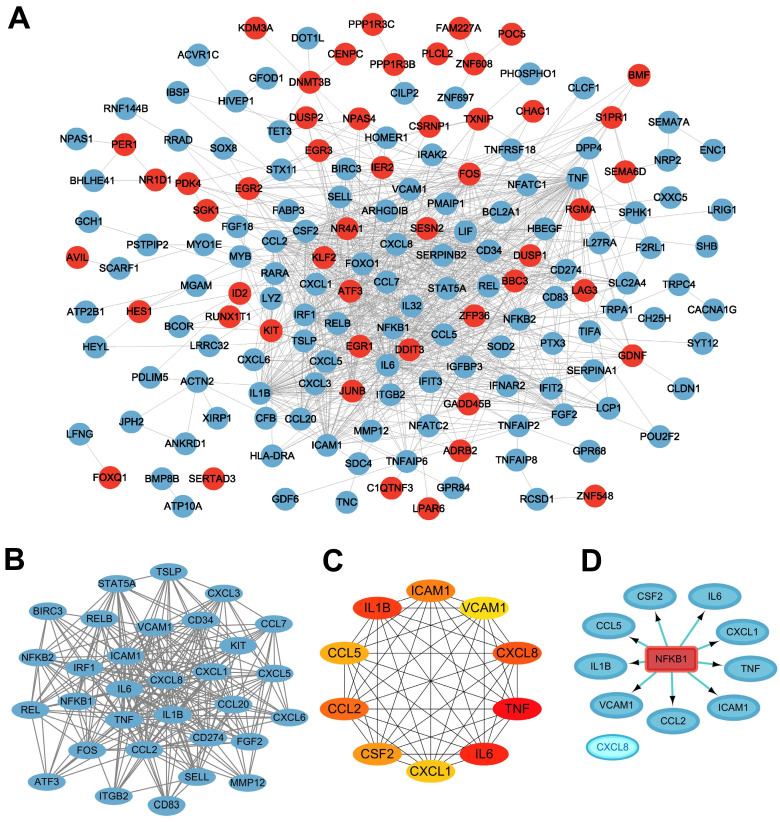
PPI network analysis and hub genes screening. (**A**) PPI network of DEGs. The red nodes represent upregulation proteins, and the blue nodes represent downregulation proteins. (**B**) The highest scoring MCODE sub-cluster. (**C**) The top 10 hub genes with the highest degree of connectivity. The red color indicates the score values, and the darker color indicates a higher score. (**D**) The master regulator transcription factor predicted by the iRegulon plug-in is highlighted in red, and the target genes are in blue.

To identify the mechanism by which cryotherapy attenuates the inflammatory status of PDLCs, we performed a PPI network analysis to explore the functional interactions of DEGs. All DEGs were analyzed using the STRING database to obtain 169 nodes and 865 edges, and the PPI network was constructed using Cytoscape software (version 3.8.1) (Figure 5A). Then, the MCODE plug-in was used to identify gene cluster modules, and one cluster module was identified which had the highest score of 18.000 with 31 nodes and 270 edges (Figure 5B). The top 10 hub genes (*TNF*, *IL1B*, *IL6*, *CXCL8*, *CCL2*, *CSF2*, *CCL5*, *CXCL1*, *ICAM1*, and *VCAM1*) (Figure 5C) were screened out by CytoHubba, a plug-in of Cytoscape. Gene expression is tightly regulated by interactions between transcription factors (TFs) and their direct target genes. Ten hub genes correlated with cryotherapy were tested for TF binding motifs using the iRegulon plug-in. The results indicated that nine genes, excluding CXCL8, were regulated by NFKB1 (Figure 5D).

### 2.6. lncRNA-miRNA-mRNA Network Analysis

**Figure 6:**
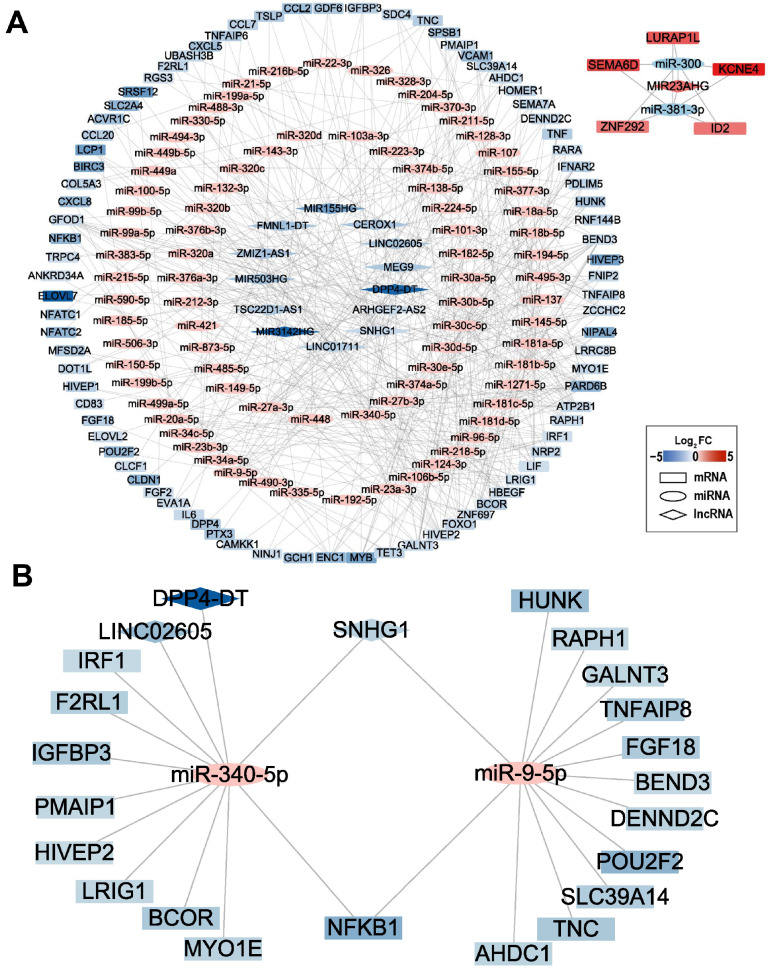
lncRNA-miRNA-mRNA network analysis. (**A**) The lncRNA-miRNA-mRNA regulatory network. The rhombuses represent lncRNAs, circles represent miRNAs, and rectangles represent mRNAs. The shade of color represents the log_2_ FC of genes, red represents upregulation, and blue represents downregulation. (**B**) The lncRNA-miRNA-mRNA network is associated with NFKB1.

To further understand the possible functions of mRNAs and lncRNAs in cryotherapy, we constructed the lncRNA-miRNA-mRNA network (Figure 6A). MiRNAs are small non-coding RNAs that regulate gene expression by binding to specific target mRNA sequences and can be regulated by lncRNAs. These RNA molecules work together to form a complex and dynamic regulatory network, namely a competitive endogenous RNA (ceRNA) network. We predicted the potential target miRNAs for the differentially expressed lncRNA, and 85 miRNAs were targeted by 14 lncRNAs. Then, a total of 87 differentially expressed mRNAs bound 85 miRNAs were predicted. There were 186 nodes and 514 edges between the 85 miRNAs, 14 lncRNAs, and 87 mRNAs.

Interestingly, we found that the lncRNA SNHG1 mediated crosstalk with NFKB1 via two miRNAs, including miR-9-5p and miR-340-5p (Figure 6B). lncRNA SNHG1 has previously been reported to be significantly associated with inflammatory gene expression and promote LPS-induced inflammatory responses [19,20,21]. Thus, we speculated that SNHG1-miR-9-5p/miR-340-5p-NFKB1 axis might play an important role in the reduction in inflammation by cryotherapy by regulating NFKB1 expression.

### 2.7. lncRNA SNHG1 Promotes LPS-Induced Inflammatory Response in hPDLCs

**Figure 7:**
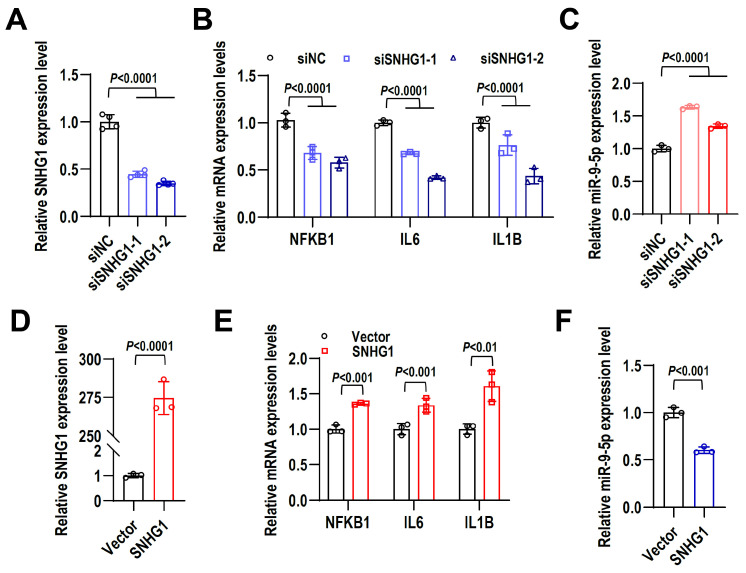
lncRNA SNHG1 promotes LPS-induced inflammatory response in hPDLCs. LPS-induced hPDLCs were used to mimic periapical inflammation for in vitro study. (**A**) Knockdown efficiency of siSNHG1-1 and siSNHG1-2 siRNAs in LPS-induced hPDLCs was confirmed by qRT-PCR. siNC is a non-specific siRNA control. (**B**,**C**) The expression levels of NFKB1, IL6, IL1B, and miR-9-5p in LPS-induced hPDLCs treated with siSNHG1 and siNC were detected by qRT-PCR. (**D**) The overexpression efficiency of lncRNA SNHG1 in LPS-induced hPDLCs was demonstrated by qRT-PCR. (**E**,**F**) The expression levels of NFKB1, IL6, IL1B, and miR-9-5p in LPS-induced hPDLCs with SNHG1 stably overexpression were examined by qRT-PCR. Data are means ± SD, *n* = 3.

To explore the function of the above potential pathways in LPS-stimulated hPDLCs, we first identified the role of SNHG1 in the inflammatory environment. We knocked down SNHG1 using siRNA in LPS-stimulated hPDLCs and detected knockdown efficiency by qRT-PCR (Figure 7A). We found that the transcriptional level of NFKB1 and proinflammatory cytokines, IL6 and IL1B, were significantly reduced, while the expression of miR-9-5p was elevated by knockdown of SNHG1 in LPS-induced hPDLCs (Figure 7B,C). Conversely, stable overexpression of SNHG1 in LPS-induced hPDLCs promoted the expression of NFKB1, IL6, and IL1B and suppressed the expression of miR-9-5p (Figure 7D–F). SNHG1 expression was negatively correlated with miR-9-5p expression while positively correlated with NFKB1. In fact, the binding sites between miR-9-5p and SNHG1 or NFKB1 have been reported [22,23]. These results suggest that SNHG1 could promote LPS-induced inflammation in hPDLCs by sponging miR-9-5p via targeting NFKB1.

### 2.8. Cryotherapy Reduces Inflammation by Regulating the lncRNA SNHG1/miR-9-5p/NFKB1 Axis

**Figure 8:**
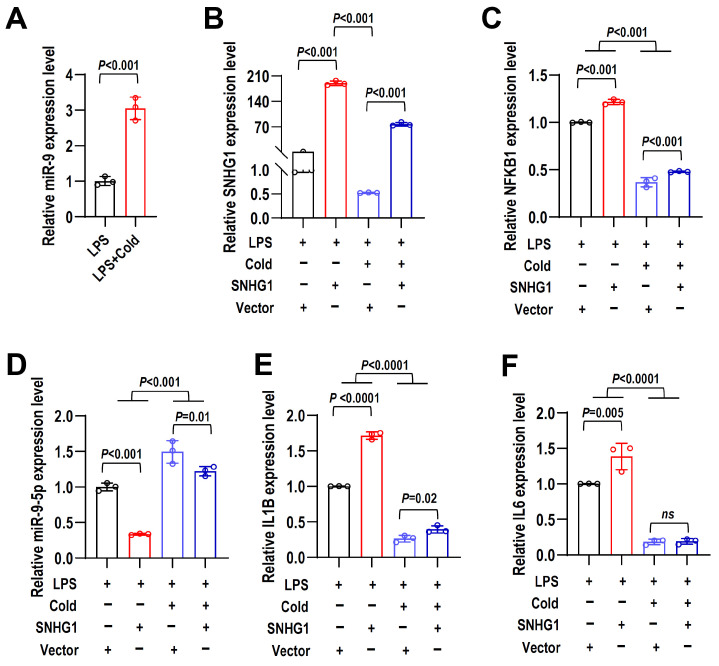
Cryotherapy reduces inflammation by regulating the lncRNA SNHG1/miR-9-5p/NFKB1 axis. (**A**) The miR-9-5p expression level in LPS-induced hPDLCs after cryotherapy was analyzed by qRT-PCR. To investigate the role of lncRNA SNHG1/miR-9-5p/NFKB1 axis in cryotherapy, hPDLCs were stably overexpressed with SNHG1 or empty vector control and then stimulated with 10 ng/mL LPS, followed by cryotherapy treatment or not. (**B**–**F**) The expression levels of SNHG1, NFKB1, miR-9-5p, IL1B, and IL6 were detected by qRT-PCR. Data are means ± SD, *n* = 3.

Next, we investigated whether cryotherapy reduced LPS-induced inflammatory responses in hPDLCs by lncRNA SNHG1/miR-9-5p/NFKB1 axis. Cryotherapy could reduce LPS-induced inflammatory response in hPDLCs, decrease the expression of SNHG1 and NFKB1 (Figure 2D,F), and increase the expression of miR-9-5p (Figure 8A). In LPS-induced hPDLCs, the overexpression of SNHG1 partially rescued NFKB1 reduction caused by cryotherapy (Figure 8B,C). Consistently, the elevated expression of miR-9-5p upon cryotherapy treatment was significantly reduced by overexpressing SNHG1 in LPS-induced hPDLCs (Figure 8D). Importantly, overexpression of SNHG1 can partially rescue the significantly reduced levels of proinflammatory cytokines IL1B induced by cryotherapy in LPS-treated hPDLCs (Figure 8E). Interestingly, after cryotherapy, IL6 expression seemed to show no difference with or without the overexpression of SNHG1 in LPS-induced hPDLCs (Figure 8F). The results indicated that lncRNA SNHG1 could act as ceRNA to increase NFKB1 expression by down-regulating miR-9-5p levels. SNHG1/miR-9-5p/NFKB1 axis is one of the pathways of cryotherapy to reduce inflammation, and the other underlying mechanisms and potential pathways remain to be explored.

## 3. Discussion

A growing number of clinical studies have indicated that cryotherapy could reduce pain after root canal therapy and may produce anti-inflammatory effects [14]. However, the mechanism of action of cryotherapy in the treatment of apical periodontitis remains unclear. In this study, we demonstrated that short-term (0.5 h) cold treatment could significantly reduce the expression level of proinflammatory cytokines, including IL1B and IL6, in LPS-induced inflammatory response in hPDLCs. Importantly, by using RNA-seq assay to analyze gene expression profiles of hPDLCs after cryotherapy, we discovered a total of 287 significantly differentially expressed genes. Differential gene expression analysis revealed that cryotherapy mainly down-regulated the expression of multiple cytokines and chemokines, which are often used as inflammatory biomarkers and associated with pathogenic mechanisms [24]. The significantly down-regulated chemokine family member genes (*CCL5*, *CXCL6*, *CXCL8*, *CCL2*, *CXCL5*, *CXCL1*, *CCL20*, *CCL7*, and *CXCL3*) were extensively involved in these processes. KEGG analysis revealed significant changes in several key pathways related to inflammation, such as the TNF signaling pathway, IL-17 signaling pathway, cytokine-cytokine receptor interaction, and NF-kappa B signaling pathway. Among them, the cytokine-cytokine receptor interaction pathway was the only common pathway for KEGG analysis and GSEA analysis, suggesting that the cytokine-cytokine receptor interaction pathway plays a key role in the response of cryotherapy to inflammatory PDLCs. In summary, our results uncover that cryotherapy may affect several inflammation-related pathways and biological processes. How these pathways and biological processes are regulated by cryotherapy requires further investigation.

To further explore the mechanism of cryotherapy, a total of 10 hub genes were screened by Cytoscape. All these hub genes have been reported to be significantly elevated in periapical inflammation and promote inflammatory responses [25,26]. The expressions of all these hub genes were downregulated after cryotherapy, and most of them (such as *TNF*, *IL1B*, *IL6*, *CXCL8*, *CCL2*, *CSF2*, *CCL5*, and *CXCL1*) are cytokines or chemokines. Furthermore, a central role of the transcription factor NFKB1 in the regulation of cryotherapy was determined. NFKB1 (p50) is produced from the cytoplasmic precursor p105 and forms a heterodimer with another NFKB subunit RelA (p65). The p50/p65 heterodimer has been proven to be the most stable and common form of NFKB and is the transcriptionally active complex in the canonical NF-κB pathway [27]. It has been reported that NF-kB played an important role in apical periodontitis and was required for the expression of many proinflammatory genes [28]. In this study, we found that cryotherapy may inhibit NFKB1 expression, which subsequently suppresses the transcription of several target genes, including proinflammatory cytokines and chemokines.

Many studies have revealed that microRNAs (miRNAs) are involved in the regulation of periapical inflammation by targeting downstream genes [29]. However, the interaction between periapical inflammation and long non-coding RNA (lncRNA) has not been reported. According to the predicted ceRNA network, we predicted that SNHG1 may be one of the target genes for cryotherapy to inhibit inflammation. Indeed, cold treatment significantly reduced SNHG1 expression, and SNHG1 is required for the expression of NFKB1, IL6, and IL1B. It has been reported that SNHG1 participates in a variety of LPS-induced inflammation and promotes the production of proinflammatory cytokines [19,20]. Interestingly, SNHG1 is a direct transcriptional target of NFKB subunit p65 that promotes the expression of proinflammatory cytokines in acute lung injury (ALI) [21]. SNHG1 may form a positive feedback loop with the NF-κB pathway.

SNHG1 expression level is positively related to NFKB1 while negatively correlated with miR-9-5p. Both SNHG1 and NFKB1 are the direct target genes of miR-9-5p. Therefore, we proposed that SNHG1 and NFKB1 mRNA could competitively bind with miR-9-5p and form a novel ceRNA regulatory pathway, SNHG1/miR-9-5p/NFKB1 axis, which may be one of the pathways of cryotherapy to reduce inflammation. Future studies are required to explore more potential molecule mechanisms.

Hypoxia exists in the apical area of inflammatory periapical lesions [30]. Hypoxia stimulates macrophages and other cells to produce proinflammatory cytokines. In the present study, we treated cells under normoxic conditions, and cells showed reduced expression of inflammatory cytokines. However, hypoxia may aggravate the inflammation reaction of cells and affect the efficiency of cold treatment. Further studies are required to explore the effect of cold treatment on inflammation under hypoxic conditions.

The main pathological bacterial species in apical periodontitis are *Fusobacterium*, *Prevotella*, *Actinomyces*, and *Streptococcus*, according to a recent report [31]. Therefore, both Gram-positive and Gram-negative bacteria can be involved in the infection of apical periodontitis. In the present study, we just checked the effects of cold treatment on the inflammatory responses induced by LPS from Gram-negative bacteria. Further studies may be required to explore the effect of cold treatment on inflammation caused by Gram-positive bacterial species, such as *Streptococcus* and *Enterococcus*.

## 4. Materials and Methods

### 4.1. Cell Isolation and Culture

Healthy human periodontal ligament tissues were collected from third molars extracted from healthy individuals aged 18 to 25 years from the Department of Oral and Maxillofacial Surgery, Hospital of Stomatology, Wuhan University. After washing each tooth with phosphate-buffered saline (PBS) solution (Hyclone, Marlborough, MA, USA), the tissue from the middle third of the root was scraped with a sterile scalpel and cut into small pieces. The tissue blocks were cultured with alpha Minimum Essential Medium (α-MEM) (Hyclone, Marlborough, MA, USA) supplemented with 10% fetal bovine serum (FBS) (Gibco, Carlsbad, CA, USA) and 1% penicillin-streptomycin (Gibco, Carlsbad, CA, USA) in 5% CO_2_ at 37 °C. The medium was changed every 3 days. Once the cell monolayer reached 80% confluence, human periodontal ligament cells (hPDLCs) were trypsinized and passaged. Cells between passages 3 and 6 were used for the subsequent experiments. For the control group, hPDLCs were cultured in an incubator at 37 °C. For cold treatment, cells were placed in a refrigerator at 4 °C. The selection of a temperature 4 °C for cold treatment was based on publications. Clinical studies used the saline solution of 2.5 °C or 4 °C cold as final irrigation in the endodontic cryotherapy to reduce postoperative pain. There was no statistically significant difference in therapeutic effect between the 4 °C and 2.5 °C treatment [15,16].

### 4.2. RNA Extraction, Reverse Transcription PCR (RT-PCR), and Quantitative RT-PCR (qRT-PCR)

Total RNA was extracted utilizing TRIzol reagent (Invitrogen, Carlsbad, CA, USA) and then reverse-transcribed into complementary DNA (cDNA) by the Maxima H Minus cDNA Synthesis Master Mix (Thermo Fisher, Waltham, MA, USA) based on the manufacturer’s instructions. The generated cDNA was used as a template in PCR amplification with Green Taq Mix (Vazyme Biotech, Nanjing, China). After PCR, the products were analyzed by running in an agarose gel, and the relative band intensity, which represents the relative expression level of the target gene, was quantified by using ImageJ software (version 1.51) [32] and normalized by the GAPDH band intensity.

Quantitative real-time PCR (qRT-PCR) was performed with the cDNA described above using ChamQ Universal SYBR qPCR Master Mix (Vazyme Biotech, Nanjing, China). GAPDH was used as an internal control for normalization, and the relative expression was calculated using the 2^−ΔΔCt^ method. At least three independent experiments were performed for statistical evaluation. The primers used in RT-PCR and qRT-PCR analysis are listed in Table S1.

MicroRNAs were purified using TRIzol reagent (Invitrogen, Carlsbad, CA, USA). The endogenous microRNA levels were analyzed using TaqMan Advanced miRNA Assays (Applied Biosystems, Carlsbad, CA, USA) in accordance with the manufacturer’s protocol. U6 was used as an internal control.

### 4.3. RNA Sequencing and DEG Identification

Total RNA was extracted from normal control or cold-treated LPS-stimulated PDLCs with TRIzol reagent (Invitrogen, Carlsbad, CA, USA) and then used for RNA sequencing by Wuhan Ruixing Biotechnology (Wuhan, China). LPS was bought from Sigma-Aldrich, USA (Cat. No. L2880). This LPS originates from *Escherichia coli* (O55:B5) and is purified by phenol extraction. The KAPA Stranded mRNA-Seq Kit (Roche, Basel, Switzerland) was used to prepare strand-specific RNA-seq libraries following the manufacturer’s instructions. A total of six cDNA libraries were high-throughput sequenced using the Illumina Novaseq 6000 system (Illumina, San Diego, CA, USA) for 150 nt paired-end sequencing. Alignment was performed against the human reference genome (GRCh38) using HISAT2 software (version 2.1.0). According to fragments per kilobase of transcript per million mapped reads (FPKM), the expression level of each gene was standardized. Differentially expressed genes (DEGs) were analyzed through the DESeq2 software (version 1.34.0). The significant DEGs were defined based on a *q*-value (adjusted *p*-value) ≤ 0.05 and absolute Log_2_ Fold Change ≥ 1. Heatmap was generated using the R package “heatmap” and volcano plot using “ggplot2” (R version 3.2.1, ggplot2 version 3.3.6).

### 4.4. Functional and Pathway Enrichment Analyses of the DEGs

Enrichment analysis was performed using the R package “ClusterProfiler” (version 4.4.4). All the DEGs were subjected to Gene Ontology (GO) functional analysis and Kyoto Encyclopedia of Genes and Genomes (KEGG) pathway enrichment analyses. GO terms and KEGG pathways with adjusted *p*-value < 0.05 were considered as significant enrichment. All bubble charts representing enrichment analysis were generated using the R package “ggplot2” (version 3.3.6). Chord plots of selected GO categories were performed using the R package “GOplot” (version 1.0.2). Pathway maps of selected KEGG pathways were visualized using the R package “Pathview” (version 1.34.0).

For gene set enrichment analysis (GSEA), all the genes were used via the R package “Clusterprofiler” and “DOSE” (version 3.20.1). Significantly enriched gene sets were selected based on nominal *p*-value (NOM *p*-value < 0.05) and false discovery rate *q*-value (FDR *q*-value < 0.25). Ridge plots of the top 10 GSEA results were generated with the R package “ggridges” (version 0.5.3) and normalized enrichment score (NES). All the GSEA analysis results were visualized using the R package “GOplot”.

### 4.5. Protein–Protein Interaction Network Analysis

The Search Tool for the Retrieval of Interacting Genes (STRING) database (http://string-db.org (accessed on 10 February 2023)) was used to construct the PPI network. The interaction combined scores greater than 0.4 were retained. The interaction nodes of the protein were analyzed and visualized by Cytoscape software (version 3.8.1). Subsequently, the Molecular Complex Detection (MCODE) was used to investigate the key gene clusters with the default parameters. The CytoHubba plug-in was used to explore hub genes. The iRegulon (version 1.3) plug-in was used to screen the key transcription factor (TF) with the default settings.

### 4.6. lncRNA-miRNA-mRNA Network Analysis

The ENCORI database was used to predict the lncRNA-targeted miRNAs and select miRNA-targeted mRNAs in DEGs based on the two different miRNA target prediction tools, including TargetScan and miRanda [33]. The lncRNA-miRNA-mRNA Network map was visualized by Cytoscape software (version 3.8.1) [34].

### 4.7. RNA Interference (RNAi) and Transfection

The small interfering RNA (siRNA) against SNHG1 and non-specific siRNA (siNC) were synthesized by Sangon Biotech (Shanghai, China). The sequences of SNHG1 siRNA are as follows: (siSNHG1-1: CCAGCAUCUCAUAAUCUAU), (siSNHG1-2: CCUUCUCUCUAAAGCCCAA). All siRNAs were transfected into cells using the Lipofectamine 8000 Reagent (Beyotime, Shanghai, China) according to the manufacturer’s protocol.

### 4.8. Plasmids and Transfection

The full-length sequence human lncRNA SNHG1 was amplified from hPDLCs and cloned into the pLVX-IRES-Puro vector (Clontech, Kusatsu, Japan) at the XhoI and BamHI sites for the construction of lncRNA SNHG1 expression plasmid. Primers used are listed in Table S2. Lentivirus was produced by co-transfecting lncRNA SNHG1 expression plasmid (or control vector) with psPAX2 and pMD2.G into HEK 293T cells using Lipofectamine 293 (Beyotime, Shanghai, China) according to the instructions provided by the manufacturer.

### 4.9. Statistical Analysis

Statistical comparison of mean values was performed using Student’s *t*-test for two groups or a one-way ANOVA test for more than two groups. Shapiro–Wilk normality test was used to analyze normal distribution, and Brown-Forsythe test was used to analyze variances. All statistical analyses were generated by GraphPad Prism8 software. The threshold for significance was set at *p*-value < 0.05.

## 5. Conclusions

In summary, we demonstrate that cryotherapy can control inflammatory responses by influencing multiple biological processes and pathways, such as SNHG1/miR-9-5p/NFKB1 axis, to reduce the expression of proinflammatory cytokines and chemokines. Our results provide a theoretical basis for the application of cryotherapy in the treatment of periapical inflammation.

## Data Availability

The RNA-seq data have been deposited in the GEO database (GSE232156).

## Supplementary Materials

The supporting information can be downloaded at: https://www.mdpi.com/article/10.3390/ijms241512097/s1.
