# Supplementary material for: Cryotherapy Attenuates Inflammation via the lncRNA SNHG1/miR-9-5p/NFKB1 Regulatory Axis in Periodontal Ligament Cells
Source: Int J Mol Sci. 2023 Jul 28;24(15):12097. doi: 10.3390/ijms241512097 (PMC10418934; doi:10.3390/ijms241512097)

Figure S1. The expression levels of IL1B and IL6 in hPDLCs with different treat. (A) The hPDLCs were either untreated (Blank) or treated with 10, 100, or 1000 ng/mL LPS for 1, 3, or 6 hours . (B) The 10 ng/mL LPS-stimulated hPDLCs were treated without or with cold treatment for 5, 10, 15, or 20 minutes. Blank is the no LPS and cold treatment control. (C) The hPDLCs were treated without or with cold treatment for 0.5, 1, or 2 hours. The expression levels of IL1B and IL6 were detected by RT-PCR.

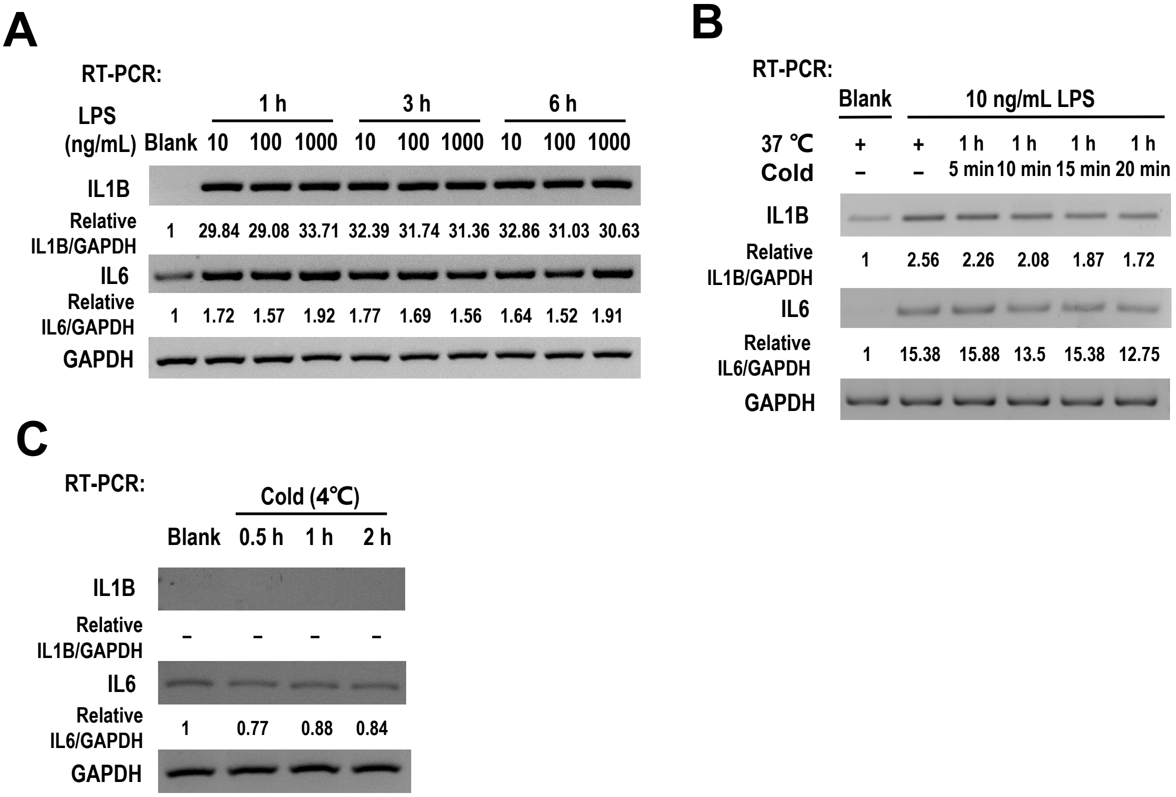

Figure S2. The GSEA analysis of the osteoclast differentiation pathway.

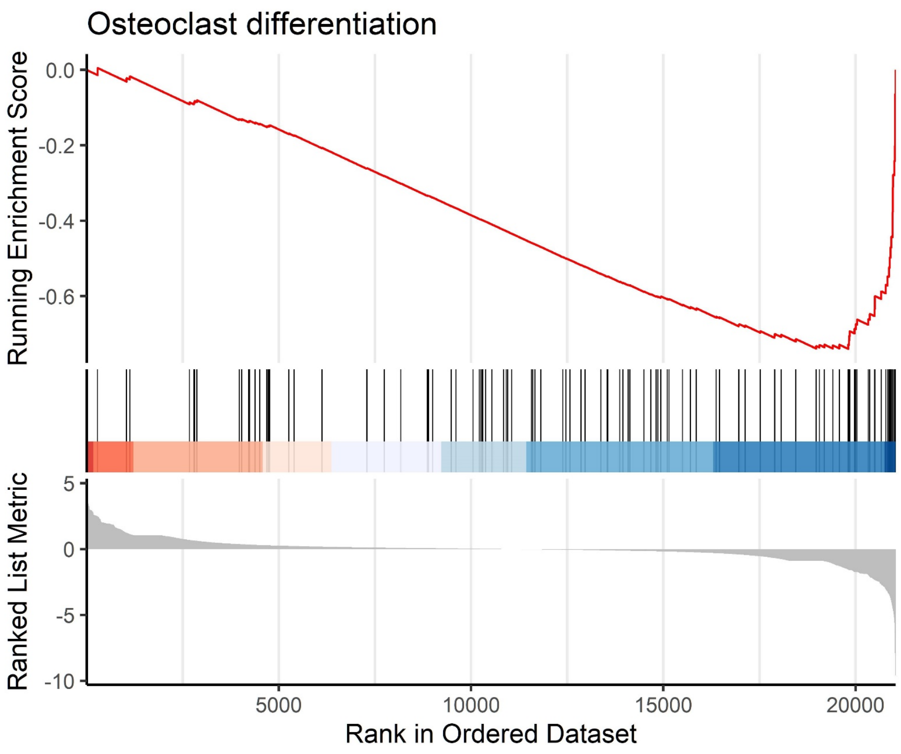

Figure S3. The KEGG pathway map of osteoclast differentiation pathway.

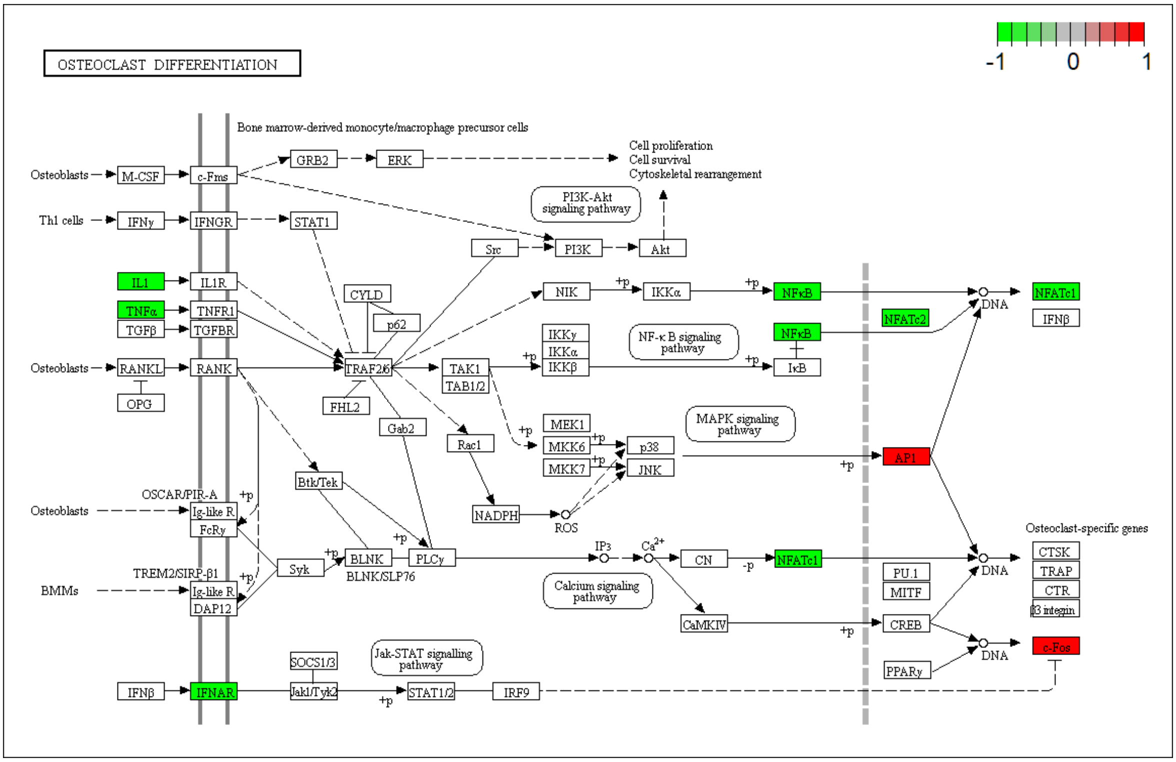

Supplement: Supplementary file 1 [file ijms-24-12097-s001.zip › Supplementary Figures.pdf]
