# Supplementary material for: Cryotherapy Attenuates Inflammation via the lncRNA SNHG1/miR-9-5p/NFKB1 Regulatory Axis in Periodontal Ligament Cells
Source: Int J Mol Sci. 2023 Jul 28;24(15):12097. doi: 10.3390/ijms241512097 (PMC10418934; doi:10.3390/ijms241512097)
Supplement: Supplementary file 1 [file ijms-24-12097-s001.zip › Supplementary Tables.pdf]

Table S1. Primers used in RT-PCR and qRT-PCR.

Gene	Forward primers (5’-3’)	Reverse primers (5’-3’)
GAPDH	GAAGGTGAAGGTCGGAGTC	GAAGATGGTGATGGGATTTC
IL6	ACTCACCTCTTCAGAACGAATTG	CCATCTTTGGAAGGTTCAGGTTG
IL1B	CTCGCCAGTGAAATGATGGCT	GGTCGGAGATTCGTAGCTGGAT
CXCL1	AGCTTGCCTCAATCCTGCATCC	TCCTTCAGGAACAGCCACCAGT
CXCL3	CCACACTCAAGAATGGGAAGA	TCTCTCCTGTCAGTTGGTGCT
CXCL5	AGCTGCGTTGCGTTTGTTTAC	TGGCGAACACTTGCAGATTAC
CXCL6	ACTTGTTTACGCGTTACGCTGAG	TTCTTCAGGGAGGCTACCACTT
CXCL8	TCTGCTAGCCAGGATCCACA	TGCTTCCACATGTCCTCACA
CCL20	TGCTGTACCAAGAGTTTGCTC	CGCACACAGACAACTTTTTCTTT
FOXO1	TCGTCATAATCTGTCCCTACACA	CGGCTTCGGCTCTTAGCAAA
TNF	CTCTTCTGCCTGCTGCACTTTG	ATGGGCTACAGGCTTGTCACTC
LIF	CCATACGCCACCCATGTCAC	GCCACATAGCTTGTCCAGGTTG
ICAM1	CCTCAGTCAGTGTGACCGCAG	GGAAAGCTGTAGATGGTCACTGTC
STAT5A	CCACAGAACCCTGACCATGTACTC	AAGACTGTCCATTGGTCGGCG
CCL2	CAGCCAGATGCAATCAATGCC	TGGAATCCTGAACCCACTTCT
NFKB1	CCTGCTGACAATTTCCCACAC	CGAGTTAAATCGAGAATGATTCAG
CD274	ATGCCTTGGTGTAGCACTGA	GCTGGATTACGTCTCCTCCAAA
VCAM1	CCGGATTGCTGCTCAGATTGGA	AGCGTGGAATTGGTCCCCTCA
SRSF12	GAAATAGGAGGCGGTCAGACAG	TGACTGCCTTGCTGAGGTAGAC
CCL5	AGCCCTCGCTGTCATCCTCA	GGCAATGTAGGCAAAGCAGC
ZFP36	GCTATGTCGGACCTTCTCAGAG	CCTGGAGGTAGAACTTGTGACAG
GADD45B	TACGAGTCGGCCAAGTTGATG	GGATGAGCGTGAAGTGGATTT
FOS	GTGGGAATGAAGTTGGCACT	CTACCACTCACCCGCAGACT
DUSP1	CCCGAGTTCCTCTGGGTTTC	CCGGATCACACACTGAGTCC
NR1D1	CTGCCAGCAATGTCGCTTCAAG	TGGCTGCTCAACTGGTTGTTGG
SESN2	AAGGACTACCTGCGGTTCG	CGCCCAGAGGACATCAGTG
RUNX1T1	CCATTGCCCACCACTA	CCACTCTTCTGCCCATT
S1PR1	CCTGTGACATCCTCTTCAGAGC	CACTTGCAGCAGGACATGATCC
ATF3	GGTTAGGACTCTCCACTCAA	AGACAGTAGCCAGCGTCCTT
NR4A1	CCAAGTACATCTGCCTGGCTAAC	CTGTTCGGACAACTTCCTTCACC
SNHG1	AGCAGACACAGATTAAGACA	GGCAGGTAGATTCCAGATAA
MIR3142HG	AGCTTGGAAGACTGGAGACAG	TCACAGGAACTCACACTCCT
CACNA1G-AS1	TTGTTGGCCGGAGCACTAAT	AGTGAAGCAGGAAGGAACCG
MIR155HG	GAGTGCTGAAGGCTTGCTGT	TTGAACATCCCAGTGACCAG

Table S2. Primers for plasmids construction.

Target	Forward primers (5'-3')	Reverse primers (5'-3')
SNHG1 #1	CTCATTTTTCTACTGCTCGTGGAT	CATCAACAACCAACACAGCAACAC
SNHG1 #2	CTCATTTTTCTACTGCTCGTGGAT	AAACAGGACTATGTAATCAATCATTT
SNHG1 #3	TTCCGCTCGAGCTCATTTTTCTACTGCTC GTGGAT	GGAGGGAGAGGGGCGGGATCCAAACAGG ACTATGTAATCAATCATTT
